# Author Correction: ITK signalling via the Ras/IRF4 pathway regulates the development and function of Tr1 cells

**DOI:** 10.1038/s41467-020-16420-4

**Published:** 2020-05-19

**Authors:** Weishan Huang, Sabrina Solouki, Nicholas Koylass, Song-Guo Zheng, Avery August

**Affiliations:** 1 Center for Clinical Immunology, The Third Affiliated Hospital of Sun Yat-sen University, Guangzhou, Guangdong 510630 China; 2 Department of Microbiology and Immunology, Cornell University, Ithaca, New York 14853 USA

Correction to: *Nature Communications* 10.1038/ncomms15871, published online 21 June 2017.

The original version of this Article contained an error in the author affiliations.

Song-Guo Zheng was incorrectly associated with the ‘Center for Clinical Immunology, The Third Affiliated Hospital of Sun Yat-sen University, Guangzhou, Guangdong 510630, China.’

This has not been corrected in the PDF and HTML versions of the Article.

