# Supplementary material for: ITK signalling via the Ras/IRF4 pathway regulates the development and function of Tr1 cells
Source: Nat Commun. 2017 Jun 21;8:15871. doi: 10.1038/ncomms15871 (PMC5482062; doi:10.1038/ncomms15871)
Supplement: Supplementary Information [file ncomms15871-s1.pdf]

Type of file: pdf
Title of file for HTML: Supplementary Information
Description: Supplementary Figures

Type of file: pdf
Title of file for HTML: Peer Review File
Description:

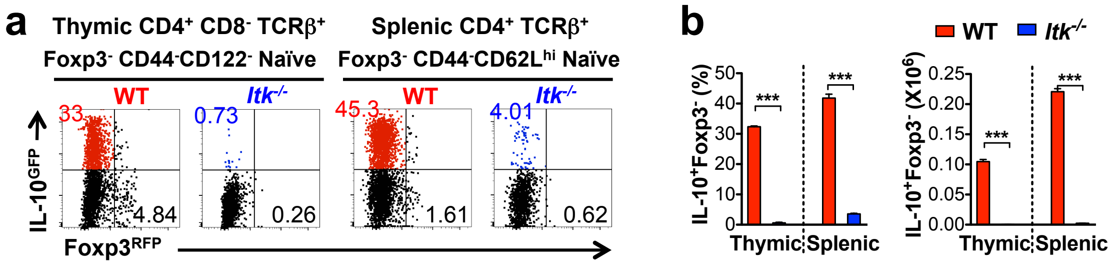

Supplementary Figure 1. ITK is required for splenic and thymic naïve $CD4^+$ T cell differentiation into Tr1 *in vitro*. Naïve WT and *Itk*$^{-/-}$ $CD4^+$ T cells were isolated from IL-10^{GFP}/Foxp3^{RFP} dual reporting mice and cultured under Tr1 polarizing conditions. (a) Representative FACS plots showing the IL-10^{GFP} and Foxp3^{RFP} expression by naïve thymic and splenic $CD4^+$ T cells cultured under Tr1 polarizing condition for 3 days. (b) Summary of Tr1 cell percentage (left) and number (#/ml, right, initial naïve $CD4^+$ T cell density: 0.5×10^6/ml). n = 6. Data represent results of 3 independent experiments using thymic cells and 20 independent experiments using splenic cells. $^{***}p \leq 0.001$ by Non-parametric Mann-Whitney test. Data presented as Mean ± S.E.M..

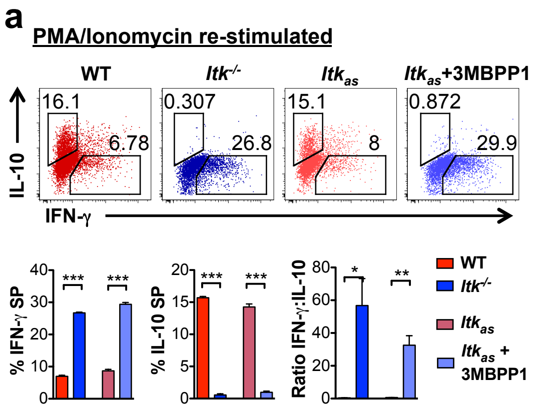

Supplementary Figure 2. ITK activity tunes the balance of IL-10/IFN-γ production by CD4$^+$ T cells. Naïve WT and *Itk*$^{-/-}$ CD4$^+$ T cells were cultured under Tr1 polarizing conditions for 60 hours, stimulated with (a) PMA/Ionomycin or (b) anti-CD3/CD28 in the presence of BFA/Monensin, and subjected to intracellular staining. Representative FACS plots for IL-10 and IFN-γ production (top), with summary of proportion and ratio of IFN-γ and IL-10 (bottom). n = 6. Data pooled from results of three experiments. $^*p \leq 0.05$, $^{**}p \leq 0.01$, $^{***}p \leq 0.001$ by Non-parametric Mann-Whitney test. Data presented as Mean ± S.E.M..

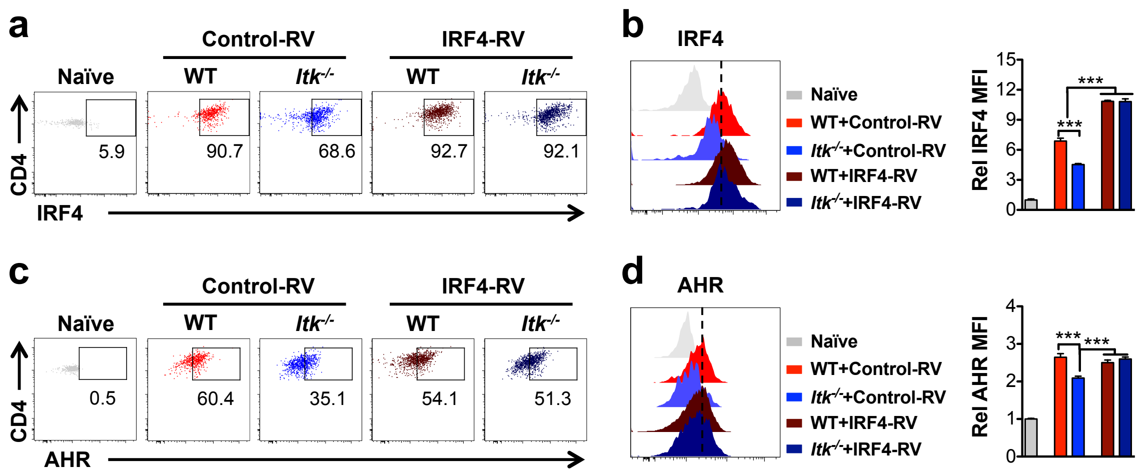

Supplementary Figure 3. Expression of IRF4 and AHR in T cells transduced with IRF4-RV. Naïve WT and *Itk*$^{-/-}$ CD4^{+} T cells were cultured under Tr1 polarizing conditions and transduced with control- or IRF4-RV. (a) Representative FACS dot plots for the expression of IRF4 in RV transduced cells. (b) Histogram showing IRF4 signal intensity (left) and summary (right) of Rel IRF4 MFI. (c) Representative FACS dot plots for the expression of AHR in RV transduced cells. (d) Histogram showing AHR signal intensity (left) and summary (right) of Rel AHR MFI. Naïve CD4^{+} T cell population in grey was used as control. n = 6. Data represent results of two experiments. $^{***}p \leq 0.001$, comparing groups connected, by Non-parametric Mann-Whitney test. Data presented as Mean ± S.E.M..

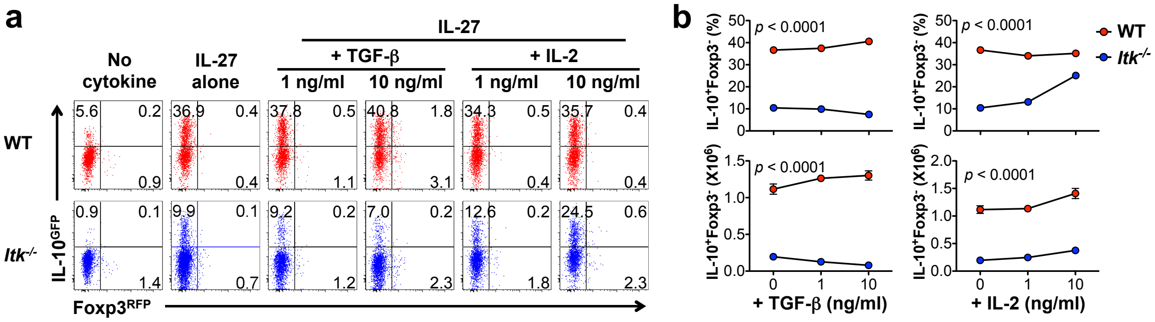

Supplementary Figure 4. The effect of TGF-β and IL-2 in WT and *Itk*$^{-/-}$ Tr1 cell differentiation. Naïve WT and *Itk*$^{-/-}$ CD4^{+} T cells were isolated from IL-10^{GFP}/Foxp3^{RFP} dual reportor mice and cultured with APCs, anti-CD3/CD28, and anti-IFN-γ/IL-12, with the cytokines indicated. (a) Representative FACS plots showing the IL-10^{GFP} and Foxp3^{RFP} expression by viable CD4^{+} T cells in the culture. (b) Summary of IL-10^{+}Foxp3^{-} Tr1 cell percentage (top) and number (bottom, initial naïve CD4^{+} T cell density: 0.5 × 10^{6}/ml), in the presence of IL-27 and various concentrations of TGF-β or IL-2. n = 4. Data represent results of 2 independent experiments. *p* values calculated by two-way ANOVA. Data presented as Mean ± S.E.M..

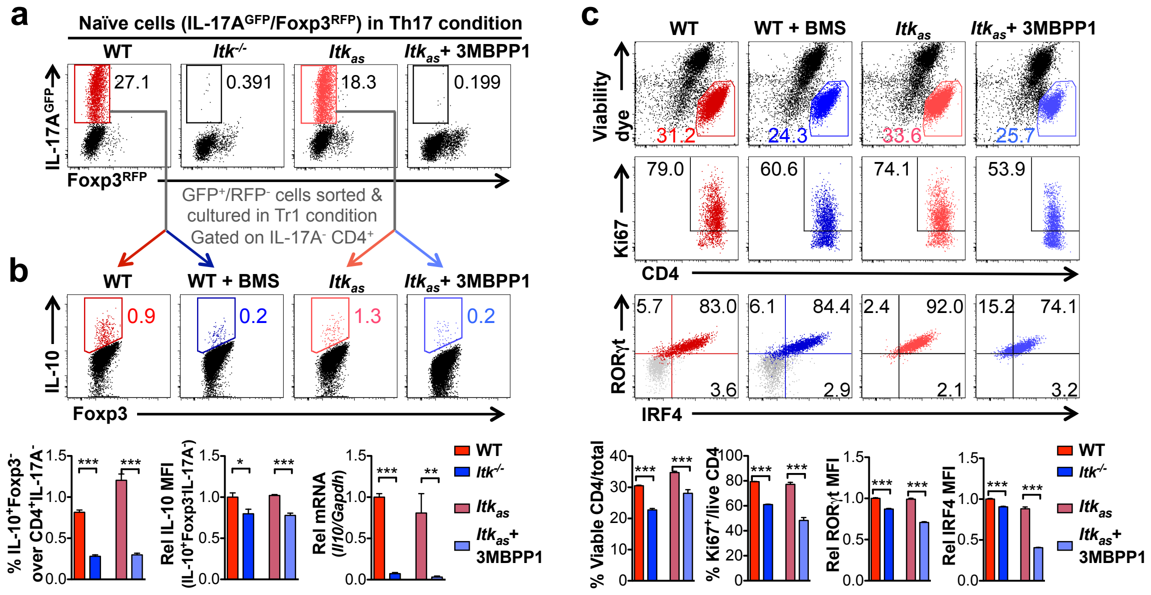

Supplementary Figure 5. ITK kinase activity regulates trans-differentiation of Th17 cells to Tr1 cells. WT and *Itk_{as}* Th17 cells were differentiated from naïve IL-17^{GFP}/Foxp3^{RFP} CD4^{+} T cells, isolated and cultured under Tr1 polarizing conditions. Cells were stimulated and subjected to intracellular staining. (a) Representative plots of Th17 cells, and (b) IL-10 and Foxp3 expression by exTh17 cell (those that lost IL-17A expression), with summary of percentage of Tr1 cells/exTh17 cells, IL-10 Rel MFI in Tr1^{exTh17} cells that are IL-10^{+}Foxp3^{-}IL-17A^{-}, and IL-10 Rel mRNA in the cultured cells. (c) Representative plots of viability, expression of proliferative marker Ki67, and transcription factor RORγt and IRF4 (naïve CD4^{+} T cells were included in the grey background as negative controls); with summary of percentage of viable CD4^{+} T cells, Ki67^{+} over viable CD4^{+} T cells, and Rel MFI of RORγt and IRF4. Data represent results of two experiments, n = 6. $^{*}p \leq 0.05$, $^{**}p \leq 0.01$, $^{***}p \leq 0.001$, by Non-parametric Mann-Whitney test. Data presented as Mean ± S.E.M..

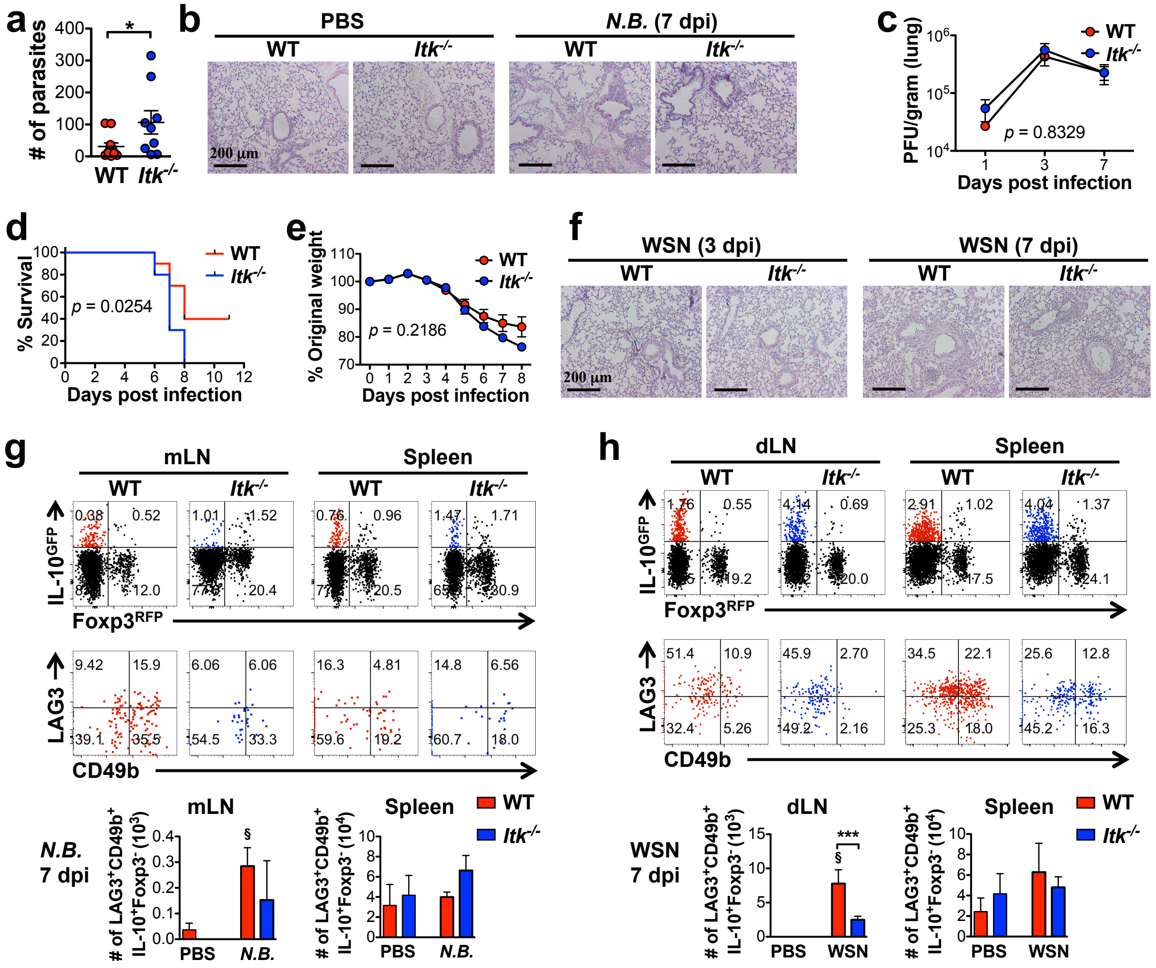

Supplementary Figure 6. WT and *Itk*$^{-/-}$ mouse responses to *Nippostrongylus brasiliensis* or Influenza A infection. WT and *Itk*$^{-/-}$ mice were infected (a, b & g) with 500 L3 *Nippostrongylus brasiliensis* (*N.B.*) and analyzed 7 days post infection (dpi), or (c-f, & h) with 10^4 PFU (LD_{50}) Influenza A/WSN/1933 (WSN) and analyzed at indicated time points. (a) Counts of *N.B.* adult parasite in the small intestine, and (b) representative histological images of WT and *Itk*$^{-}$ mouse lungs 7 dpi of *N.B.*. (c) Plaque-forming units (PFU) in lungs collected from WSN infected mice at indicated time points. n = 6. (d) Survival of WT and *Itk*$^{-/-}$ mice infected with WSN. n = 10, at the initial time point. p value was calculated by Log-rank test. (e) Percentage of original weight of WT and *Itk*$^{-/-}$ mice infected with WSN. n = 10, at the initial time point. p value was calculated by two-way ANOVA. (f) Representative histological images of WT and *Itk*$^{-}$ mouse lungs 3 and 7 dpi of WSN. (g & h) Representative FACS plots for IL-10 vs. Foxp3 (top) and LAG3 vs CD49b (middle) expression, with summary of number of Tr1 cells (bottom) in lymph nodes (LN) and spleen of mice 7 dpi of (g) *N.B.* or (h) WSN. mLN: mesenteric LN; dLN: draining LN. n = 4. $^{\S}p \leq 0.05$, compared to the basal level in PBS-treated group; $^{*}p \leq 0.05$, $^{***}p \leq 0.001$, comparing groups connected, by Non-parametric Mann-Whitney test. Data presented as Mean ± S.E.M..

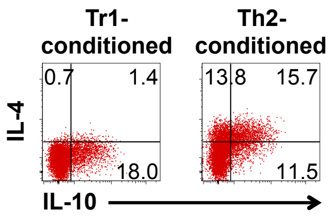

Supplementary Figure 7. Low level of IL-4 production by the induced Tr1 cells under Tr1-polarizing condition. Naïve $CD4^+$ T cells isolated from $IL\text{-}10^{GFP}/Foxp3^{RFP}$ dual reporter mice were cultured under Tr1 and Th2 cell differentiating conditions for 3 days, then stimulated by PMA/Ionomycin/BFA/Monensin for the last five hours. Representative plots of IL-10/IL-4 expression (by intracellular cytokine staining) in $CD4^+$ T cells cultured under Tr1-polarizing or Th2-polarizing conditions. Data represent results of two independent experiments.

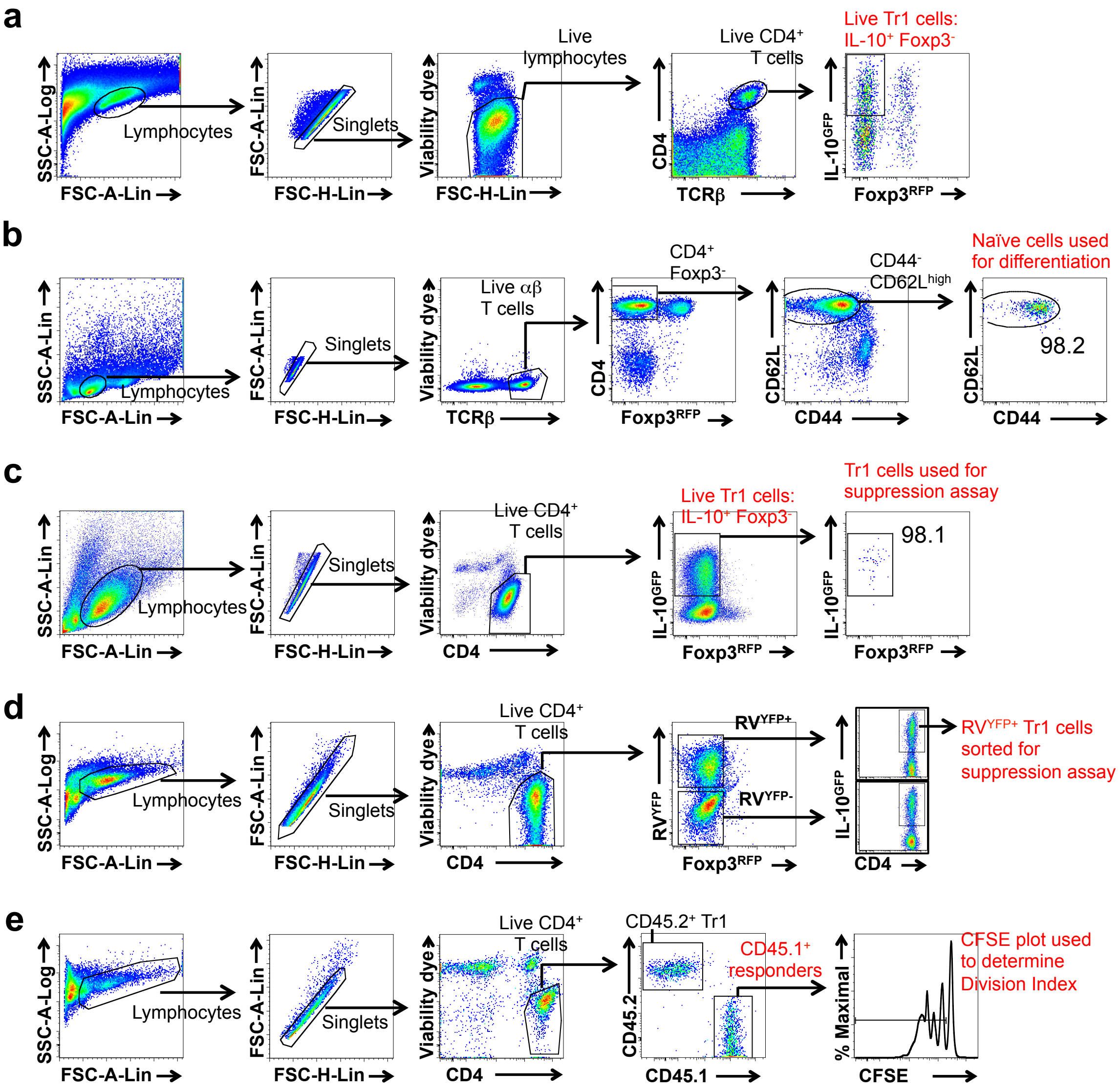

Supplementary Figure 8. Gating strategies for cell sorting and analyses. Representative FACS plots showing: (a) Gating strategy to identify viable CD4^{+} T cells that are IL-10^{GFP+} Foxp3^{RFP-} Tr1 cells from cells freshly isolated from tissues. (b) Gating strategy for isolating naïve CD4^{+} T cells by flow sorting for *in vitro* T cell differentiation. (c) Gating strategy to analyze T cells differentiated *in vitro* and to isolate these cells by flow cytometry for functional assays. (d) Gating strategy to analyze T cells transduced with retroviral particles during T cell differentiation *in vitro*. (e) Gating strategy to analyze Tr1 cell suppressive ability.
